# Probing the Dynamics of *Streptococcus pyogenes* Cas9 Endonuclease Bound to the sgRNA Complex Using Hydrogen-Deuterium Exchange Mass Spectrometry

**DOI:** 10.3390/ijms23031129

**Published:** 2022-01-20

**Authors:** Polina V. Zhdanova, Alexander A. Chernonosov, Daria V. Prokhorova, Grigory A. Stepanov, Lyubov Yu. Kanazhevskaya, Vladimir V. Koval

**Affiliations:** 1 Institute of Chemical Biology and Fundamental Medicine, Siberian Branch of the Russian Academy of Sciences (SB RAS), 630090 Novosibirsk, Russia; p_chalova@niboch.nsc.ru (P.V.Z.); alexander.chernonosov@niboch.nsc.ru (A.A.C.); prohorova1994@gmail.com (D.V.P.); stepanovga@niboch.nsc.ru (G.A.S.); lyubov.kanazhevskaya@niboch.nsc.ru (L.Y.K.); 2 Department of Natural Sciences, Novosibirsk State University, 630090 Novosibirsk, Russia

**Keywords:** HDX-MS, hydrogen-deuterium exchange mass spectrometry, CRISPR–Cas systems, Cas9, single guide RNA, molecular dynamics

## Abstract

The Cas9 endonuclease is an essential component of the CRISPR–Cas-based genome editing tools. The attainment of high specificity and efficiency of Cas9 during targetted DNA cleavage is the main problem that limits the clinical application of the CRISPR–Cas9 system. A deep understanding of the Cas9 mechanism and its structural-functional relationships is required to develop strategies for precise gene editing. Here, we present the first attempt to describe the solution structure of Cas9 from *S. pyogenes* using hydrogen-deuterium exchange mass spectrometry (HDX-MS) coupled to molecular dynamics simulations. HDX data revealed multiple protein regions with deuterium uptake levels varying from low to high. By analysing the difference in relative deuterium uptake by apoCas9 and its complex with sgRNA, we identified peptides involved in the complex formation and possible changes in the protein conformation. The REC3 domain was shown to undergo the most prominent conformational change upon enzyme-RNA interactions. Detection of the HDX in two forms of the enzyme provided detailed information about changes in the Cas9 structure induced by sgRNA binding and quantified the extent of the changes. The study demonstrates the practical utility of HDX-MS for the elucidation of mechanistic aspects of Cas9 functioning.

## 1. Introduction

A Cas9 endonuclease from *Streptococcus pyogenes* is a part of the type II CRISPR–Cas adaptive immune system, which aims to protect bacterial cells against foreign DNA [1,2]. In association with a specific guide RNA (sgRNA), Cas9 generates a double-strand break (DSB) in the target DNA close to a protospacer adjacent motif PAM (5′-NGG in *S. pyogenes*). The resulting DSB can be further processed by one of two DNA repair pathways: high-fidelity homologous recombination (HDR) or error-prone nonhomologous end joining (NHEJ) [3]. Repair of DSBs through NHEJ leads to incorporation of random insertions/deletions, resulting in a frameshift and gene knockout, as long as HDR provides a precise genome modification using a homologous repair template. Today, a number of CRISPR–Cas-based genome editing technologies are rapidly evolving, although much remains to be done to achieve the desired efficiency and specificity of these tools. Clinical applications of CRISPR biology are constrained by the necessity of reducing an off-target activity of Cas endonucleases [4,5]. Strategies to increase the system’s specificity include the development of ‘high-fidelity’ variants of Cas9 by directed evolution, structure-guided engineering, and RNA guide engineering [6,7,8,9]. Success in meeting these challenges is directly related to a clear understanding of structure-function relationships in the Cas9–sgRNA–DNA complex.

**Figure 1:**
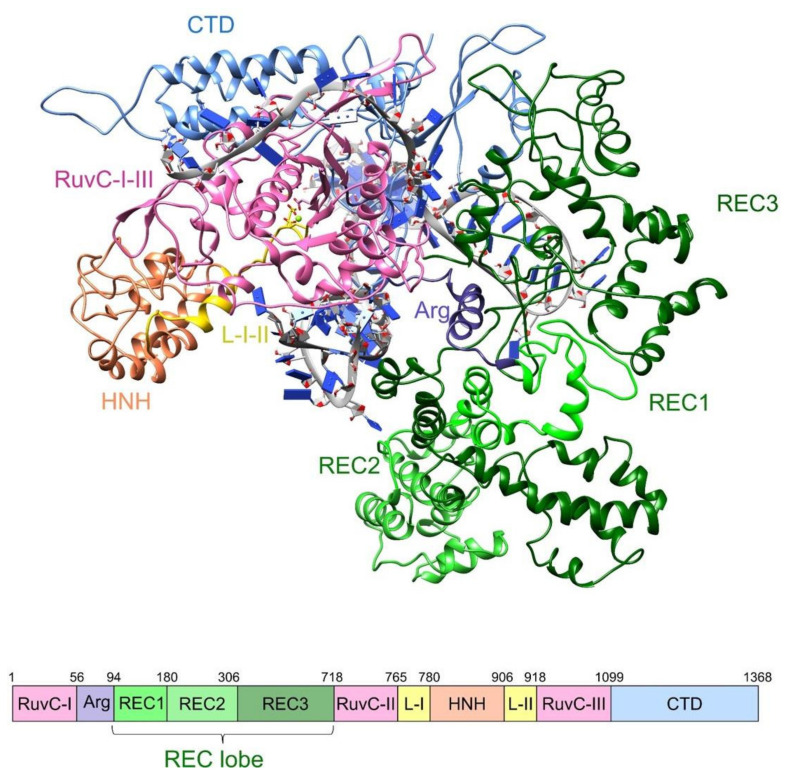
The overall structure of Cas9 from *S. pyogenes* complexed to sgRNA and a target DNA (PDB ID 4CMQ) [11]. Each protein domain is coloured according to the domain organization diagram below the structure.

The multidomain structure of Cas9 from *S. pyogenes* gives rise to its multifunctional nature (Figure 1). Besides its function in the direct RNA-guided recognition and cleavage of target DNAs, this enzyme also participates in crRNA maturation and spacer acquisition [10]. The Cas9 protein consists of an α-helical recognition REC lobe and a nuclease NUC lobe, connected by an Arg-rich bridge helix [11]. The NUC lobe comprises the conserved RuvC and HNH domains, which are structurally similar to nuclease domains presented in other endonuclease families. In particular, a ββα-metal fold of HNH shares structural homology with the phage T4 endonuclease VII and cleaves the target DNA strand through a single-metal mechanism [12]. RuvC, in turn, has a retroviral integrase RNase H fold to attack the scissile phosphate in the target DNA strand through the two-metal mechanism [13]. A C-terminal region of the NUC lobe, the so-called CTD domain, contains other structural elements involved in interactions with the RNA–DNA heteroduplex, such as the PAM-interacting domain (PI domain) and a ββαβ topoisomerase II-like domain (Topo-homology domain) [11,14]. The REC lobe consists of three α-helical domains (REC1, REC2, and REC3), which are the least conserved across the Cas9 orthologs within the Type II CRISPR system and are not similar to any other known protein domains [14]. Data obtained by cryo-EM microscopy and X-ray crystallography for pre-catalytic, post-catalytic, and product-bound states of Cas9 indicate that bringing the apoprotein into the catalytically active state requires a significant rearrangement of its conformation, especially in the REC lobe and HNH domain [11,15]. Specifically, the binding of sgRNA by the HNH, REC1, and Arg-helix domains results in the formation of a central channel structure for target DNA binding. The following recognition of the PAM sequence by hydrogen-bonding interactions with two Arg residues located in the PI domain and local melting of the DNA duplex facilitate the emergence of a cleavage-competent state. The PAM-induced correlated motions in the RuvC and HNH domains most likely regulate the concerted target DNA cleavage through an allosteric mechanism [16,17]. This mechanism has been further clarified by molecular dynamics simulations in a micro-to-millisecond time range, suggesting that the highly cooperative conformational dynamics of the REC1, REC2, REC3, and HNH domains provide the catalytic competence of the system as a whole [18]. The X-ray and cryo-EM structures have demonstrated the considerable conformational flexibility of Cas9, wherein some parts of the structure, including the PI domain and L1 linker, are disordered in the absence of a hybrid heteroduplex [11,14,15,19,20]. MD simulations have also revealed that the microsecond conformational dynamics of Cas9 domains from an ‘open’ to a ‘closed’ state promote specific nucleic acids’ binding and processing [18,21]. Further studies by single-molecule FRET have shed more light on the Cas9 functional dynamics in solution [22,23].

Hydrogen–deuterium exchange mass-spectrometry (HDX-MS) is a powerful analytical technique to study protein conformation and dynamics in solution [24,25,26]. This approach is especially useful for studying large or weakly structured proteins that are not amenable to X-ray crystallography. The rate of deuterium exchange in protein amide hydrogens is sensitive to the solvent accessibility and conformation of the protein structural elements, providing a dynamic picture of protein structure. CRISPR–Cas systems operate as huge nucleoprotein complexes comprising large multidomain enzymes with many disordered elements, which complicates the building of its atomic structure. Therefore, HDX-MS could be a promising tool for elucidating solution phase protein conformation and dynamics. To date, the only protein of the CRISPR-associated complex, Cas3 nuclease-helicase, has been described by HDX-MS [27]. Here, we used HDX-MS analysis coupled to molecular dynamics simulation to probe the structural dynamics of apoCas9 and the Cas9–sgRNA complex in solution. It was found that almost seventy peptides of Cas9 are subjected to the hydrogen-deuterium exchange. Comparing the deuterium uptake profiles from free and RNA-bound states revealed protein regions involved in the sgRNA binding. The findings testify to the high potential of the HDX-MS method for elucidating the solution structure and dynamics of large molecular weight proteins such as Cas9.

## 2. Results

### 2.1. Molecular Dynamics

Matching the hydrogen-deuterium exchange behaviour with the Cas9 structural domains requires a full-length structural model of the protein. Most of the existing crystal structures of Cas9 and its complexes are represented as truncated sequences or contain unresolved disordered regions [11,28]. For this reason, we decided to carry out a computer modelling of the full-length apoCas9, Cas9–sgRNA, and Cas9–sgRNA–DNA complex structures. To obtain the initial coordinates of all atoms, we conducted a homology modelling of Cas9 using the Phyre2 server. The most appropriate homolog structure has been shown to be Cas9 from *Streptococcus pyogenes* with the PDB ID: 4CMQ [11]. To provide the relevant model, we supplemented it with the missing residues using Chimera 1.15 and the Modeller software. Mg^2+^ ions and the sgRNA–DNA heteroduplex were manually added to the initial model. Initial coordinates for the heteroduplex were derived from the crystal structure of the Cas9–sgRNA–DNA complex from *Staphylococcus aureus* (PDB ID: 5CZZ) by adding and modifying nucleotides using LEaP (Amber20) and UCSF Chimera 1.15. The initial theoretical model included 1360 amino acids, 85-mer sgRNA, 10-mer DNA, and three Mg^2+^ ions. It should be noted that nucleotide sequences in the modelled complex structure differed from experimental sequences used for HDX analysis (sgRNA—103 nt, DNA—33 nt), but the key elements of both sequences were still maintained.

**Figure 2:**
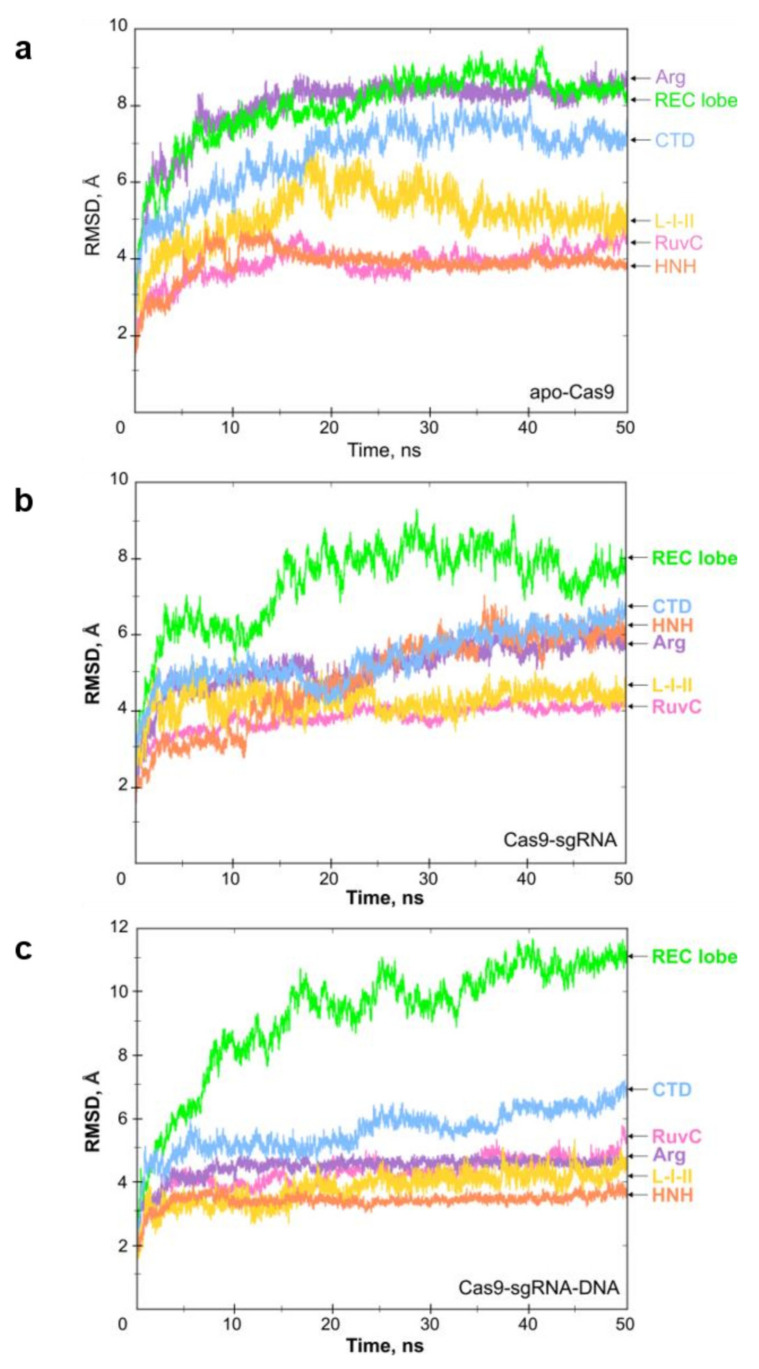
Time evolution of RMSD values (in angstroms, *Y* axis) calculated in 50 ns trajectories (*X* axis) for apoCas9 (**a**), Cas9–sgRNA complex (**b**)**,** and Cas9–sgRNA–DNA ternary complex (**c**). The average RMSD values for the REC lobe (green), CTD (blue), RuvC (pink), and HNH (orange) domains, L-I-II (yellow), Arg (violet), linkers are shown. The deviation amplitude correlates with the protein dynamic behaviour.

The results of MD simulations give information about the stability of the free protein, its complexes and its dynamic behaviour. The conformational dynamics were quantified with an energy landscape of RMSD values (Figure 2). We analysed the RMSD values for the main domains of Cas9. The apoprotein showed relative stability throughout the MD trajectory (50 ns). As follows from Figure 2a, all protein domains reached equilibrium quickly (~5 ns) and fluctuated within 1 Å for the rest of the simulation time.

The time evolution of the RMSD values for the complex Cas9–sgRNA revealed good stability for all domains except for the REC lobe, whose fluctuation behaviour substantially changed during interactions with sgRNA (Figure 2b). After the intermittent growth of the RMSD up to 8 Å during the first 18 ns, fluctuations of the REC domain stabilized between 7 and 9 Å. The maximum fluctuations of the NUC lobe components were within 6.2 Å. Specifically, the average RMSD values of the RuvC and HNH domains were ~4.0 Å and ~6.2 Å, respectively. The RMSD of the CTD domain (~4.8 Å) was stable over 8 ns, but after that, it increased to ~6.2 Å.

The RMSD profile for simulation of the ternary complex Cas9–sgRNA–DNA generally corresponded to that of the Cas9–sgRNA complex. However, the addition of DNA substrate to the system influenced the character of fluctuations of specific domains (Figure 2c). Indeed, the NUC lobe element HNH (orange) and the Arg bridge helix (violet) exhibited a higher level of stability through all dynamics simulations as compared to the simulation of the Cas9–sgRNA complex. The maximum fluctuations in the NUC lobe components were within 7 Å. In particular, the average RMSD values for RuvC and HNH were ~5 and ~3.4 Å, respectively. The REC lobe and CTD domains demonstrated a lower stability level. The molecular dynamics of the REC lobe were characterized by a gradual increase in the RMSD value up to ~11.5 Å. By considering the RMSD values of individual components of the REC lobe (REC1, REC2, and REC3), we found these domains to fluctuate in different ways. The average RMSD values of REC1 and REC2 domains were ~7.2 and ~5.1 Å, respectively. In comparison, the RMSD of REC3 achieved ~10–11 Å, substantially contributing to the observed instability of the REC lobe (data not shown). Thus, our data established the most mobile domain of REC as REC3. This result agrees with the earlier findings that demonstrated a ~65 Å shifting of REC3 toward the HNH domain upon Cas9 binding to sgRNA [16].

**Figure 3:**
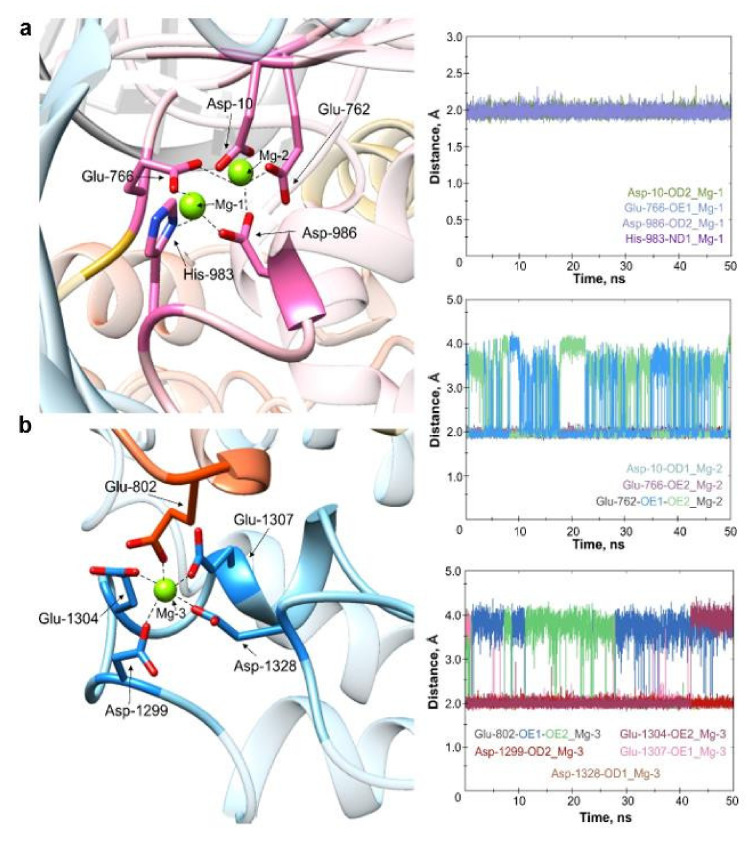
MD simulations of the metal coordination centres in the Cas9–sgRNA–DNA complex. (**a**) Coordination of metal ions (Mg-1, Mg-2, green spheres) by amino acid residues of the RuvC domain (pink) of Cas9. (**b**) Localization of Mg^2+^ (Mg-3, green sphere) within the CTD domain (blue). The coordinating residue Glu-802 belongs to the HNH domain (orange). Graphs on the right demonstrate fluctuations in the distance (RMSD) between the metal and functional groups of its coordinating amino acids in the course of MD simulation.

According to its domain structure, the Cas9 protein should contain four Mg^2+^ cations: two ions in the RuvC nuclease domain, one in the HNH nuclease domain, and one in the CTD domain. However, crystal structures often lack adequate coordinates for metal ions. Our MD simulations applied the coordinates of the metal ions derived from the crystal structure 4CMQ, which is relevant to the coordinating amino acid residues. The modelled structures of apoCas9 and its complexes with sgRNA and DNA involved three Mg^2+^ ions residing in the RuvC and CTD domains. Figure 3 represents the distance between each metal and the coordinating amino acids of Cas9 in the Cas9–sgRNA–DNA complex. The position of both Mg^2+^ ions within the RuvC domain was persistent throughout the MD trajectory. The metals were found to be coordinated by the carboxylate moieties of Asp-10, Glu-762, Glu-766, Asp-986, and His-983 (Figure 3a) at equal distances (~2 Å), which is in agreement with the crystallographic data [11]. During simulation, we observed that only one oxygen atom of the Glu-762 carboxylate moiety was involved in coordination of the metal at a particular time. Thus, the distance between the coordinating oxygen and Mg^2+^ was maintained at ~2 Å, while another oxygen moved away by ~4 Å (see the middle graph in Figure 3 for details). The third Mg^2+^ ion (Mg-3) was located between the CTD and HNH domains. Asp-1299, Glu-1304, Glu-1307, and Asp-1328 from the α-helical PAM-interacting domain and Glu-802 from HNH provide coordination of the Mg-3 ion (Figure 3b). The side chain of Glu-802 demonstrated the “switching” of its carboxyl oxygens throughout the MD trajectory similarly to Glu-762, as described above (see the bottom graph in Figure 3 for details). During the last 10 ns, the distance between the carboxyl moiety of Asp-1328 and Mg-3 increased up to ~4 Å. Overall, the stability of the metal ions’ position during modelling demonstrates the relevance of the whole complex model presented. We used the UCSF Chimera to produce a molecular movie to visualize the system’s dynamic behaviour (Supplementary Movie S1).

### 2.2. HDX-MS

We began by drawing up a peptide map of the Cas9 protein. For this purpose, the protein solution was equilibrated at 21 °C for 1 h, and an aliquot was added to the quench buffer, imitating conditions of the exchange reaction. The protein was further digested with the pepsin immobilized on a column over 2 min. The resulting peptides were separated by an analytical column and analysed by LC–MS. The peptide coverage of the protein created using Proteome Discoverer 2.2 was 64% (Supplementary Figure S1). Due to its high reproducibility, this peptide map can be further used for quantitative analysis of deuterium exchange data.

**Figure 4:**
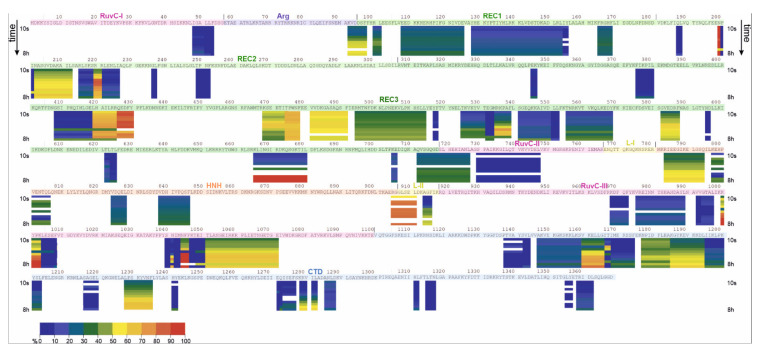
The deuteration map for the apoCas9 protein from *S. pyogenes*. The bars are coloured according to the relative percent deuterium incorporation as indicated in the legend from a dark blue to red colour. Deuterium uptake between 10% and 30% was considered ‘low’, uptake between 31% and 70% as ‘medium’, and uptake between 71% and 90% as ‘high’. A time-dependent growth of the uptake level was considered as ‘gradual’. For each peptide, the deuteration level was calculated for each of the experimental HDX incubation periods and is shown by the arrows left and right of each protein segment: 0.17, 0.5, 1, 2, 5, 10, 30, 60, 120, 240, 360, 480 min.

To examine the structure of apoCas9 in solution, the hydrogen-deuterium exchange of the protein was carried out at 21 °C. The exchange reaction was terminated at the time points of 10 s, 30 s, 1 min, 2 min, 5 min, 10 min, 30 min, 60 min, 120 min, 240 min, 360 min and 480 min by addition of the quench buffer. MS analysis of the samples was carried out after the protein digestion on the pepsin column. The HDExaminer 3.0.3 program was applied to process the raw MS data. All the spectra were manually validated to improve the accuracy of their processing. The deuterium incorporation was expressed as relative fractional uptake. Figure 4 represents a heat map of the experimentally observed hydrogen exchange in 68 identified peptides of apoCas9 over time (a high-resolution version of Figure 4 can be found in Supplementary Materials). HDX-MS analysis revealed protein regions that are relatively resistant to HDX and regions that exchange more rapidly. We divided all obtained peptides into four groups according to their exchange levels and rates (Table 1). The first group comprises peptides with less than 30% of deuterium uptake. These regions with the most buried amide positions most likely lie inside the protein globule and are inaccessible to the solvent. In the case of apoprotein, the most buried peptides are generally located in the REC2, REC3, RuvC III, and CTD domains. One peptide each was detected in domains RuvC I-II and REC1. The second group contains peptides from RuvC II-III, REC1-3, CTD, and HNH with a medium exchange level that changes little with time. Peptides from the third group demonstrated rapid and high HDX with a stable exchange level during 8 h of monitoring. This behaviour is typical to unstructured regions such as loops and other elements exposed to the protein surface. According to our data, these poorly ordered peptides represent loops and α helical elements from the REC2 domain and L-II linker connecting HNH and RuvC. Peptides from the last group exhibited a gradual hydrogen exchange corresponding to the protein regions with an explicit secondary structure. This group represented α-helix regions presumably situated on the globule surface. It includes peptides from the REC2, REC3, RuvC III, and CTD domains, and the linkers L-II and Arg, located on the surface of the RNA-binding cleft. Notably, our HDX experiment detected a gradual uptake for peptide 92–97, which forms part of the Arg α helical linker. Thus, our results indicate that deuterium uptake levels of different degrees occur in all domains and interdomain linkers of apoCas9, except for L-I. To visualize the process of deuterium uptake over time, we superimposed the script derived from HDExaminer over the PDB structure 4CMQ (Supplementary Movie S2).

**Figure 5:**
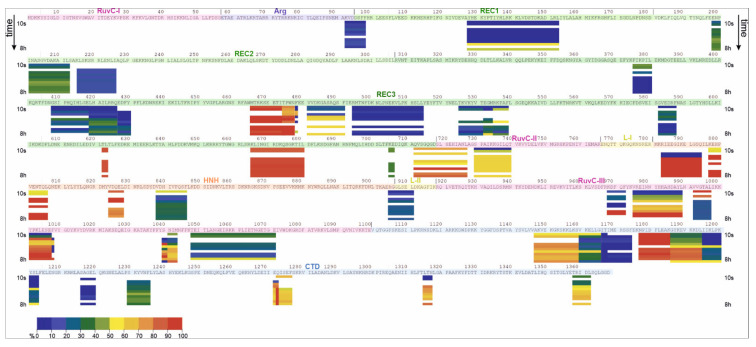
The deuteration map for the Cas9–sgRNA complex. The bars are coloured according to the relative percent deuterium incorporation as indicated in the legend from a dark blue to red colour. Deuterium uptake between 10% and 30% was considered ‘low’, uptake between 31% and 70% as ‘medium’, and uptake between 71% and 90% as ‘high’. A time-dependent growth of the uptake level was considered as “gradual”. For each peptide, the deuteration level was calculated for each of the experimental HDX incubation periods and is shown by the arrows left and right of each protein segment: 0.17, 0.5, 1, 2, 5, 10, 30, 60, 120, 240, 360, 480 min.

Next, we carried out HDX-MS for Cas9 bound to the guide RNA molecule. The protein–RNA complex (1:10 molar ratio) was formed immediately before the exchange reaction. The Cas9–sgRNA complex displayed the uptake of deuterium, albeit the amount of detected peptides (42) was lower than in the case of apoCas9 (Figure 5, a high-resolution version can be found in Supplementary Materials). Similar to apoCas9, the obtained peptides were divided into groups with low, medium, high, and gradient deuterium uptake levels. The deuterium exchange behaviour for the Cas9–sgRNA complex and apoprotein were then compared (Table 1). The findings indicated a substantial change in the conformation of certain domains during the transition of Cas9 from the free to the bound state. Differences in deuterium uptake by the same peptides in different enzyme states were analysed using the following allowance. If the peptide in the bound state showed an increase in the deuterium uptake level over the free form, it was highlighted with one of the red tints in Table 1 (see Supplementary Notes for additional information). The peptides which displayed decreased exchange levels in the Cas9–sgRNA complex were highlighted with one of the blue tints. As a result, it was determined that nine peptides reduce their availability for deuterium, and twenty-one peptides increase the deuteration level upon sgRNA binding. Overall, peptides with pronounced differences in fractional uptake were found in the RuvC II, RuvC III, REC2, REC3, HNH, and CTD domains as well as in the L-II and Arg linkers, suggesting extensive changes in their conformation, flexibility, and/or solvent exposure upon binding to sgRNA.

**Figure 6:**
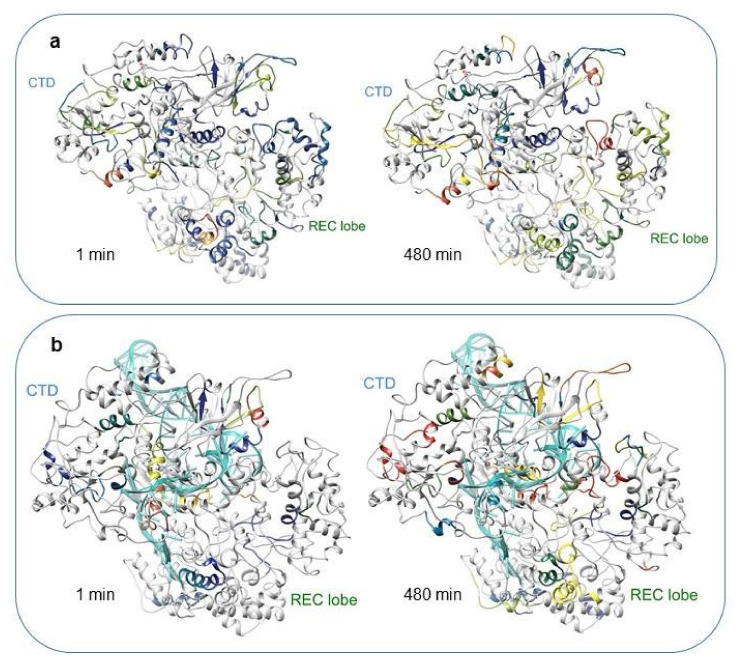
Relative deuterium uptake by apoCas9 (**a**) and Cas9-sgRNA (**b**) at the time points of 1 min and 480 min superimposed on the structure obtained from MD trajectories. RNA is shown as a ribbon in light-blue colour. The colour of peptides matches that in the corresponding deuteration map (Figure 4 and Figure 5).

Currently, HDX-MS data cannot be directly transformed into the atomic resolution structures, so the best way to interpret these results is to superimpose them onto available static structures (X-ray, MD). To analyse the distribution of the deuterium uptake across the protein structure, the measured relative deuterium uptake after 1 and 480 min of incubation in D_2_O was projected onto our simulated structures for the Cas9–sgRNA complex (Figure 6 (a high-resolution version can be found in Supplementary Materials), Supplementary Movie S3). We also mapped HDX peptides onto the available crystal structures 4CMQ [11] and 4ZT0 [28], supplemented by unresolved disordered loop regions using Modeller software (see the Supplementary Chimera Session S1). The results demonstrated a good correlation between high or low uptake levels and protein areas exposed to or protected from water. In particular, peptide 92–97, located on the edge of the interdomain linker Arg, is illustrative of the exchange behaviour in a free vs. bound state. This α-helical linker lies on the inner surface of the helical lobe cleft [11] and makes extensive contacts with sgRNA. According to the HDX data, the deuterium uptake by the peptide 92–97 in the bound state was changed from gradual to low, suggesting strong protection of its amide hydrogens from the exchange by sgRNA. This assumption is supported by the decreasing RMSD values observed when switching between the apoCas9 and Cas9–sgRNA model structures (Figure 2a,b). A similar tendency was observed for the L-II linker region representing a loop with α-helical elements (peptide 905–911). In apoCas9, this peptide is situated on the protein surface, whereas in the complex, it is surrounded by loop regions of the RuvC III and HNH domains, resulting in a reduction in the exchange level from high to low (Supplementary Chimera Session S1). This result is aligned with the findings obtained by cryo-EM microscopy for the catalytically competent ternary complex of Cas9, where the L-II linker undergoes a helix-to-loop conformational change to facilitate the displacement of the HNH domain [15].

Exchangeable peptides detected in domains RuvC II and III increased the deuterium uptake levels by a greater or lesser extent upon binding to sgRNA. It is likely that the conformations of these domains were changed substantially toward less ordered state, resulting in increased amide hydrogen accessibility to the solvent. According to our MD data, interactions between the α-helix 719–727 and nearby nucleotides G11-C14 of the sgRNA protospacer region led to helix-to-loop transformation followed by an increase in deuteration (Supplementary Chimera Session S1 for UCSF Chimera 1.15 and Chimera X). Notably, peptide 730–740 appeared to interact with the phosphodiester backbone of sgRNA close to nucleotides C71 and U72 and increase the exchange level. Four peptides with a medium uptake level were identified in the other NUC lobe domain HNH of the apoCas9 structure. The process of Cas9–sgRNA complex formation increased the solvent accessibility for at least three of these peptides. 

Domains of the REC lobe are widely represented on the deuteration map, whereas the largest number of peptides was registered in the REC3 domain (Table 1). The sequence of REC1 contains peptides with low and medium exchange levels that do not change between the free and bound states, which points to the relative conformational stability of the domain. The heat map of the REC2 domain is predominated by peptides with a low uptake level. The only peptide that changed its deuteration level from gradual to low upon sgRNA binding was the loop region 202–212 situated on the protein surface. In apoCas9, the exchange level of its amide protons slightly grew from 40 to 60%, increasing the exposure time. Formation of the Cas9–sgRNA complex resulted in a reduction in the exchange level of up to 30–50%, which was likely due to the shielding of this peptide by other structural elements of the protein (Supplementary Chimera Session S1).

In terms of structural dynamics, the most exciting domain of the REC lobe is REC3. The analysis of MD trajectories implies that the REC3 domain made a significant contribution to the high RMSD values (Figure 2a,b), which denote a high level of mobility. HDX-MS demonstrated an extensive coverage of REC3 by peptides of different exchange levels and rates, many of which changed the accessibility for the solution environment in the bound state. Some peptides were immersed inside, and some reached the protein surface. Indeed, loop motifs 426–430, 494–514, 532–539, 582–587, and α-helix 532–539 showed medium and high uptake levels in the free state, but in the bound state, their uptake levels were reduced because these peptides were immersed into the protein globule and contacted RNA structure. On the other hand, there were peptides (465–477, 622–623, 665–680, and 712–718) in the REC3 domain that increased the availability for exchange in the bound state. For example, peptide 465–477 was engaged in intermolecular contacts with nucleotides 56–58 of the repeat–antirepeat RNA region and got closer to the surface. The C-terminal region (peptide 712–718) of REC3 represented by the loop and α helical motifs is considered the linker connecting the REC and NUC lobes [16]. Peptide 712–718 showed a medium exchange level in the apoprotein, but it transformed into a peptide with a high and fast exchange in the Cas9–sgRNA complex. It can be seen from our model structure that the displacement of the REC lobe in the course of sgRNA binding results in the eversion of the peptide onto the globule surface (Figure 6, Supplementary Chimera Session S1). Therefore, the HDX and MD data pool indicate a high level of mobility and dynamic behaviour for the REC3 domain conformation upon interactions with sgRNA.

Regarding the CTD domain, the model and X-ray data suggest the predominance of loops connected by short α-helical and β-fold motifs, as well as its general surface arrangement. Thus, one would expect an efficient hydrogen–deuterium exchange in the sequence. However, according to the HDX data, most of peptides detected in the CTD domain of apoCas9 demonstrated a low uptake level, although some of them were located close to the protein surface. As the experimental conditions were designed to minimize or completely exclude the back exchange, we can speculate about the possible difference in the solid and solution structures of CTD. This could be related to protein–protein interactions or protein oligomerization in solution; however, additional experiments are needed to corroborate this. Another contributing factor for the decreased exchange rates for the exposed regions may be the presence of structured water molecules near the amino acid backbone [25]. In the course of Cas9–sgRNA complex formation, a noticeable rearrangement in the CTD domain conformation was observed, resulting in a substantial increase in the exchange efficiency by peptides 1147–1159, 1271–1276 and a medium increase by peptides 1177–1185, 1315–1317, 1358–1362. These sites are likely to become more accessible to the solvent due to their moving out from the surrounding structural elements. At the same time, the reduced deuterium uptake level in the bound state by peptide 1160–1165 (β sheet) is consistent with the model structure of the complex, where it indirectly participates in coordinating the stem-loop of sgRNA.

## 3. Materials and Methods

### 3.1. Purification of the Cas9 Protein 

A full-length Cas9 protein was expressed and purified according to the previously described protocol [29]. The pMJ806 plasmid that encodes His-tagged Cas9 was transformed into Rosetta 2 DE3 cells (Merck, Kenilworth, NJ, USA). The expression was induced by adding 0.2 mM IPTG and shaking at 18 °C overnight. After centrifugation, the lysate was loaded onto a metal-chelating IMAC resin (BioRad, Hercules, CA, USA). The protein of interest was eluted by a buffer containing 250 mM imidazole. Fractions containing Cas9 were pooled and treated with TEV protease to remove the His-tag. The protein sample was then dialyzed against 20 mM HEPES-KOH (pH 7.5), 150 mM KCl, 1 mM DTT, and 1 mM EDTA using dialysis tubes with MWCO of 12–14 kDa at 4 °C overnight. The extent of protease cleavage was controlled by SDS-PAGE. The Cas9 protein was further purified by size exclusion chromatography on the 16/600 Superdex 200 pg column (GE Healthcare, Chicago, IL, USA) in a buffer containing 20 mM HEPES-KOH (pH 7.5), 500 mM KCl, 1 mM DTT. Peak fractions were concentrated using a 30 kDa MWCO centrifugal concentrator Amicon (Merck, Kenilworth, NJ, USA) Aliquots of 50 μL were flash-frozen in liquid nitrogen and stored at −80 °C.

The enzymatic activity of the purified Cas9 was controlled according to the protocol described in [30], except for the conditions of the reaction quenching. In brief, 8 nM of target or non-target plasmid DNA of 6 or 7 kbp lengths, respectively, was incubated with the purified Cas9 protein (200 nM) and sgRNA (200 nM) for 1 h at 37 °C in a cleavage buffer (20 mM HEPES pH 7.5, 150 mM KCl, 0.5 mM DTT, 0.1 mM EDTA, 10 mM MgCl_2_). The reactions were quenched by adding a buffer containing proteinase K (60 mM EDTA, 4 M Urea, 0.4 mg/mL proteinase K), and samples were incubated for 15 min at 37 °C. The products were analysed by agarose gel (1%) electrophoresis with the EtBr staining and visualized by a UV imager (Supplementary Figure S2).

### 3.2. Preparation of the Cas9–sgRNA Complex

The 103-nt sgRNA was in vitro transcribed with T7 polymerase and purified by 10% denaturing polyacrylamide gel electrophoresis. The sequence of sgRNA contained a 5′-terminal 20-nt guide region (underlined):

5′ GGUUGGACAUGCUCGACAUU CGUUUUAGAGCUAGAAAUAG-40

CAAGUUAAAAUAAGGCUAGUCCGUUAUCAA-80

CUUGAAAAAGUGGCACCGAGUCGGUGCUUUUUU-3′

Reconstitution of the Cas9–sgRNA complex for HDX-MS was conducted by incubating Cas9 and guide RNA in a 1:10 molar ratio in a buffer containing 20 mM HEPES-NaOD (pD 7.6), 150 mM NaCl, 1 mM TCEP, and 20 mM Mg^2+^. The concentration of Cas9 for HDX-MS experiments with the protein–RNA complex was chosen to be sufficiently above the previously measured *Kd* value [31].

### 3.3. Computer Simulation

Firstly, the 4107-nt sequence of the Cas9 gene was translated to a corresponding peptide sequence using EMBOSS Transeq (Hinxton, Cambridgeshire, UK) [32]. Then, a homology modelling of the Cas9 structure was performed by the Phyre2 server [33]. As a result, the structure of Mn^2+^-bound *Streptococcus pyogenes* Cas9 (PDB ID: 4CMQ, [11]) was assigned as a reference with the identity of 92%. The alignment procedure was performed in Chimera 1.15 (San Francisco, CA, USA) [34]. To obtain the complete structure of Cas9, we used the Modeller software (San Francisco, CA, USA) [35]. To place the sgRNA and target DNA molecules within the initial Cas9–sgRNA–DNA complex, specific coordinates of the nucleic acids were extracted from the crystal structure of the *Staphylococcus aureus* Cas9–sgRNA–DNA complex (PDB ID: 5CZZ) [19]. This complex included an 85-nt sgRNA and a 10-nt DNA substrate. Finally, we positioned the Mg^2+^ ion within the complex structure according to data described by Jinek et al. [11].

Molecular dynamics (MD) simulation was conducted in an implicit solvent model using Amber 20 (San Francisco, CA, USA) [36] with GPU acceleration, the ff14SB force field for the protein, the OL3 and bsc1 force fields for the RNA and DNA, respectively, and the TIP3P water force field for the magnesium ions. Subsequently, heating of the system proceeded gradually from 20 K to 300 K during 100 ps. The equilibration step followed by production simulation took place in implicit solvent for 50 ns at 300 K in a constant pressure ensemble. Every 1 ps, the algorithm saved the system’s state (atom coordinates) to study the structural dynamics of the enzyme–substrate complex. UCSF Chimera software provided molecular graphics, MD movies, and analyses.

### 3.4. HDX-MS

Samples of Cas9 protein were prepared in a buffer containing 20 mM HEPES-NaOH (pH 7.6), 150 mM NaCl, pH 7.6, 1 mM TCEP, using the 30 kDa Amicon concentrator. Using 1 mM TCEP ensures that the complex has fully reduced SH-bridges during the labelling. Before adding the deuterated buffer, all solutions were equilibrated for 1 h at room temperature. After equilibration, Cas9 was subjected to isotopic labelling by incubating it with a deuterated buffer (20 mM HEPES-NaOD (pD 7.6), 150 mM NaCl, 1 mM TCEP) at room temperature, so that the final concentration of the protein was 0.6 μM. The total deuterium content in the labelling solution was ~90%, thus ensuring that the kinetics favoured unidirectional exchange. The reaction was stopped by the addition of an ice-cold quench buffer (2.5% formic acid, 4 M urea, pH ~2.5) to aliquots at various times (10 s, 30 s, 1 min, 2 min, 5 min, 10 min, 30 min, 60 min, 120 min, 240 min, 360 min, 480 min). The final quenched solution contained ~30% deuterium and 10 pmol of Cas9. The samples were immediately snap-frozen in liquid nitrogen and stored at −70 °C. Triplicate analyses were performed for each time point and condition for all HDX-MS analyses. The Cas9–sgRNA complex was prepared with a 10-fold molar excess of the guide RNA. The complex was stabilized by adding Mg^2+^ ions to the deuterated buffer up to 20 mM. The RNA solution was added immediately before the deuterated buffer was added. The deuterium exchange protocol was similar to that for the free Cas9 protein.

### 3.5. LC-MS Analysis

Samples were rapidly thawed prior to the mass spectrometric analysis. We performed proteolytic digestion (Enzymate BEH pepsin column, NovaBioAssays, Woburn, MA, USA) and peptide trapping/desalting (ACQUITY UPLC R Peptide BEH C18 VanGuard pre-column; 300 Å, 1.7 μm, 2.1 × 5 mm; Waters, Milford, MA, USA) with 0.15% formic acid in H_2_O (Solvent A) at 100 μL/min for 2 min, online. To separate peptides, we used (ACQUITY UPLC R Peptide BEH C18 analytical column; 300 Å, 1.7 μm, 1 × 100 mm; Waters) an 8 min linear gradient from 5% to 30% of Solvent B (100% ACN, 0.1% formic acid, pH 2.5) at 40 μL/min followed by a 2 min linear gradient from 30% to 40% of Solvent B. Then, the column was successively washed by 95% of Solvent B and 100% of Solvent A several times. After each run, the pepsin column was manually washed by a solution of 1% formic acid, 5% acetonitrile, and 1.5 M guanidinium chloride (pH 2.5) twice. Blank injections (0.15% formic acid in H_2_O) were performed between each sample to confirm the absence of carry-over. The LC flow was directed to an HF QExactive mass spectrometer (Thermo Fischer Scientific, Waltham, MA, USA) equipped with a standard electrospray ionization (ESI) source. Mass spectra were acquired in a positive-ion mode over the m/z range of 200–3000 Da. The MS scans were carried out using the Full MS—ddMS2 template.

### 3.6. Data Analysis

The identification of peptides was performed by processing the raw data on the Proteome Discoverer (v 2.2, Thermo Fischer Scientific) using a user-defined database containing the Cas9 sequence (see Supplementary Materials). Further filtration of the identified peptides from raw files by the HDExaminer software (version 3.0.3, Thermo Fischer Scientific) resulted in 64% coverage of the Cas9 sequence. No adjustment was made for a back-exchange level, and the results are reported as relative deuterium exchange levels expressed in either mass unit or fractional exchange as described elsewhere [24].

## 4. Conclusions

The present study is the first attempt to probe the structure of Cas9 from *S. pyogenes* in solution by the HDX-MS approach. To obtain an adequate full-length model of the free and RNA-bound state of Cas9, MD simulations were performed. The full-length protein structure containing especially poorly ordered and loop regions was found to facilitate the exchange behaviour interpretation. Matching results of both techniques clearly show that solvent accessibility in the model structure correlated with the observed relative deuterium uptake. In particular, peptides resistant to HDX from domains RuvC II-III and REC2-3 lie inside the structure and are surrounded by other protein motifs. In contrast, peptides with medium uptake levels observed in HNH, RuvC III, and REC1-3 are primarily situated on the protein surface. We have found that the binding of the guide RNA caused substantial conformational changes in domains RuvC III, REC3, HNH, and CTD, and linkers Arg and L-II, and less prominent changes in the REC1-2 and RuvC II domains. Our results demonstrate that the REC3 domain has a high level of plasticity, which is considered a ‘sensor’ of the complete RNA:DNA hybrid formation [18]. Thus, HDX-MS analysis complements available solid structures with a dynamic profile of individual Cas9 domains in solution. Considering the apoCas9 and Cas9–sgRNA complex without the target DNA made it possible to distinguish specific peptides exclusively involved in interactions with sgRNA. This dynamically captured structure and accompanying functional analyses have uncovered the tiny details of the mechanism of RNA–directed DNA targeting with Cas9, thereby paving the way for the rational design of new, versatile genome editing techniques.

## Figures and Tables

**Table 1:** The deuterium uptake behaviour for detected peptides of apoCas9 and the Cas9–sgRNA complex distributed amongst the protein domains. Differences in fractional uptake between apoCas9 and Cas9–sgRNA are shown in a progressive scale of red and blue colours. The lowest percent relative difference is shown in a light colour, and the highest difference is shown in a dark colour.

	apoCas9 (Free State)				Cas9-sgRNA (Bound State)			
Domain	Low uptake (<30%)	Medium uptake (30–70%)	High uptake (70–90%)	Gradual uptake	Low uptake (<30%)	Medium uptake (30–70%)	High uptake (70–90%)	Gradual uptake
RuvC I	48–53							
RuvC II	730–747	719–727				730–740	719–727	
RuvC III	968–972 975–989 992–997 1004–1008 1039–1043 1047–1050	1051–1071		1000–1003, 1044–1046	1048–1071		992–997	975–989
					968–972			999–1008
								1038–1043

Arg				92–97	92–97			
REC1	128–155	99–101 108–125 164–168			128–153			
REC2	6 peptides between a.a. 187 and 418	202–212		220–221	216–226	198–212		
					220–221			

REC3	346–347 375–380 408–418 4 peptides between a.a. 517 and 596 622–625 705–706	468–488 481–491 494–514 582–587 712–718	419–425 426–430 474–478	535–539 555–568, 582–587 665–680	375–380	532–539	465–477	481–491
					408–418	705–706	622–623	
					419–430		665–680	
					494–514		712–718	
					525–531			
					582–587			
L-II			905–911	914–916	905–911			
HNH		783–794 797–806 824–828 838–846				838–846	783–794 797–806 824–828	
CTD	1137–1144 1147–1159 1166–1174 1195–1203 1217–1220 1241–1242 1271–1276 1285–1288 1311–1312 1315–1317 1355–1356 1358–1362	1177–1184		1160–1165 1185–1194 1228–1235	1166–1174 1195–1203 1216–1220	1160–1165 1229–1235	1147–1159	1186–1194
							1177–1185	1315–1317 1358–1362
							1271–1276	
	

## Data Availability

DOI 10.5281/zenodo.5833530.

## Supplementary Materials

The following are available online at https://www.mdpi.com/article/10.3390/ijms23031129/s1.
