# Supplementary material for: Probing the Dynamics of Streptococcus pyogenes Cas9 Endonuclease Bound to the sgRNA Complex Using Hydrogen-Deuterium Exchange Mass Spectrometry
Source: Int J Mol Sci. 2022 Jan 20;23(3):1129. doi: 10.3390/ijms23031129 (PMC8834707; doi:10.3390/ijms23031129)
Supplement: Supplementary file 1 [file ijms-23-01129-s001.zip › ijms-1522751_Supplementary_materials.pptx]

## Slide 1
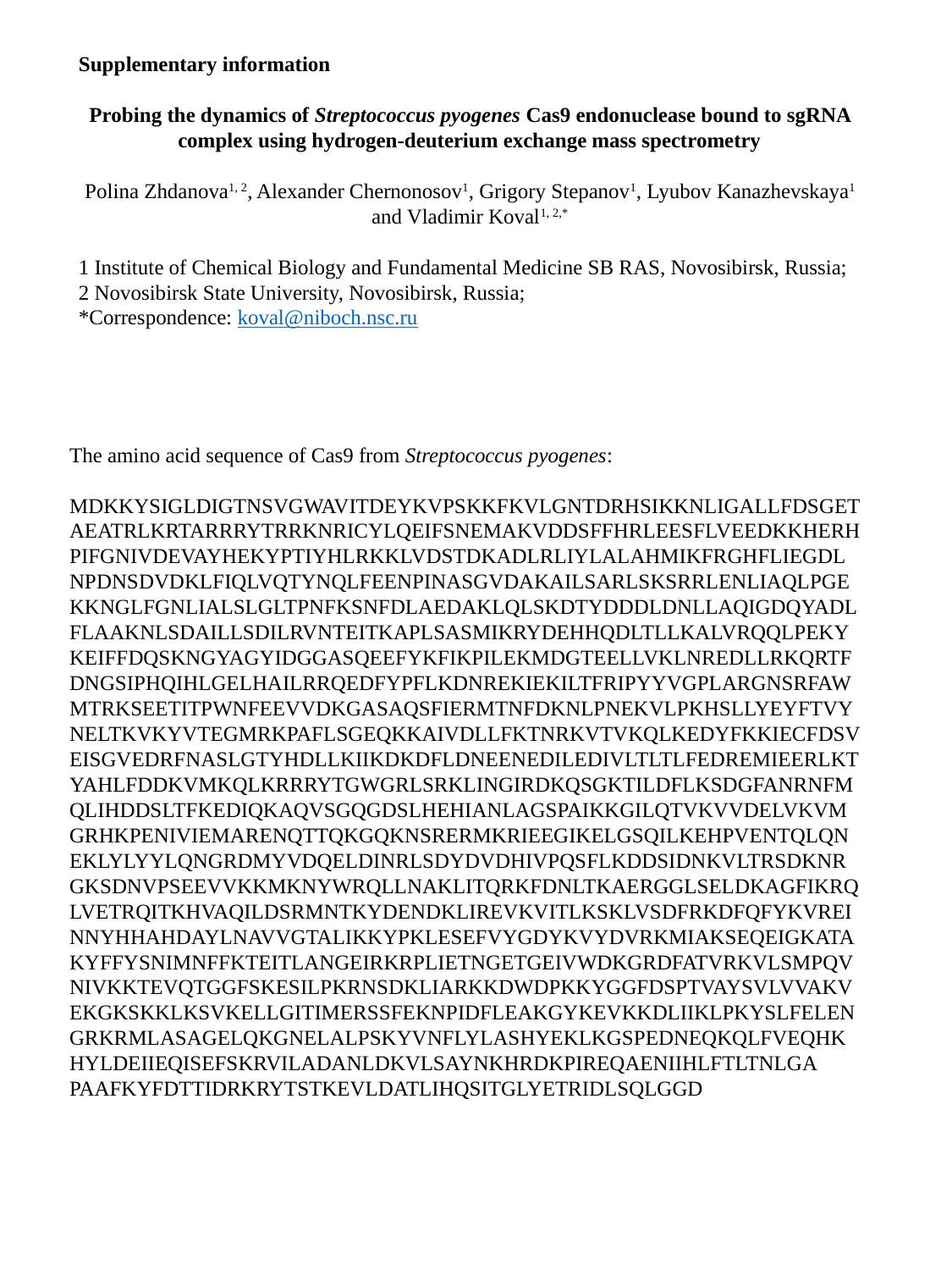

Supplementary information
Probing the dynamics of Streptococcus pyogenes Cas9 endonuclease bound to sgRNA complex using hydrogen-deuterium exchange mass spectrometry
Polina Zhdanova1, 2, Alexander Chernonosov1, Grigory Stepanov1, Lyubov Kanazhevskaya1 and Vladimir Koval1, 2,*
1 Institute of Chemical Biology and Fundamental Medicine SB RAS, Novosibirsk, Russia;
2 Novosibirsk State University, Novosibirsk, Russia;
*Correspondence: koval@niboch.nsc.ru
The amino acid sequence of Cas9 from Streptococcus pyogenes:
MDKKYSIGLDIGTNSVGWAVITDEYKVPSKKFKVLGNTDRHSIKKNLIGALLFDSGETAEATRLKRTARRRYTRRKNRICYLQEIFSNEMAKVDDSFFHRLEESFLVEEDKKHERHPIFGNIVDEVAYHEKYPTIYHLRKKLVDSTDKADLRLIYLALAHMIKFRGHFLIEGDLNPDNSDVDKLFIQLVQTYNQLFEENPINASGVDAKAILSARLSKSRRLENLIAQLPGEKKNGLFGNLIALSLGLTPNFKSNFDLAEDAKLQLSKDTYDDDLDNLLAQIGDQYADLFLAAKNLSDAILLSDILRVNTEITKAPLSASMIKRYDEHHQDLTLLKALVRQQLPEKYKEIFFDQSKNGYAGYIDGGASQEEFYKFIKPILEKMDGTEELLVKLNREDLLRKQRTFDNGSIPHQIHLGELHAILRRQEDFYPFLKDNREKIEKILTFRIPYYVGPLARGNSRFAWMTRKSEETITPWNFEEVVDKGASAQSFIERMTNFDKNLPNEKVLPKHSLLYEYFTVYNELTKVKYVTEGMRKPAFLSGEQKKAIVDLLFKTNRKVTVKQLKEDYFKKIECFDSVEISGVEDRFNASLGTYHDLLKIIKDKDFLDNEENEDILEDIVLTLTLFEDREMIEERLKTYAHLFDDKVMKQLKRRRYTGWGRLSRKLINGIRDKQSGKTILDFLKSDGFANRNFMQLIHDDSLTFKEDIQKAQVSGQGDSLHEHIANLAGSPAIKKGILQTVKVVDELVKVMGRHKPENIVIEMARENQTTQKGQKNSRERMKRIEEGIKELGSQILKEHPVENTQLQNEKLYLYYLQNGRDMYVDQELDINRLSDYDVDHIVPQSFLKDDSIDNKVLTRSDKNRGKSDNVPSEEVVKKMKNYWRQLLNAKLITQRKFDNLTKAERGGLSELDKAGFIKRQLVETRQITKHVAQILDSRMNTKYDENDKLIREVKVITLKSKLVSDFRKDFQFYKVREINNYHHAHDAYLNAVVGTALIKKYPKLESEFVYGDYKVYDVRKMIAKSEQEIGKATAKYFFYSNIMNFFKTEITLANGEIRKRPLIETNGETGEIVWDKGRDFATVRKVLSMPQVNIVKKTEVQTGGFSKESILPKRNSDKLIARKKDWDPKKYGGFDSPTVAYSVLVVAKVEKGKSKKLKSVKELLGITIMERSSFEKNPIDFLEAKGYKEVKKDLIIKLPKYSLFELENGRKRMLASAGELQKGNELALPSKYVNFLYLASHYEKLKGSPEDNEQKQLFVEQHKHYLDEIIEQISEFSKRVILADANLDKVLSAYNKHRDKPIREQAENIIHLFTLTNLGA
PAAFKYFDTTIDRKRYTSTKEVLDATLIHQSITGLYETRIDLSQLGGD

## Slide 2
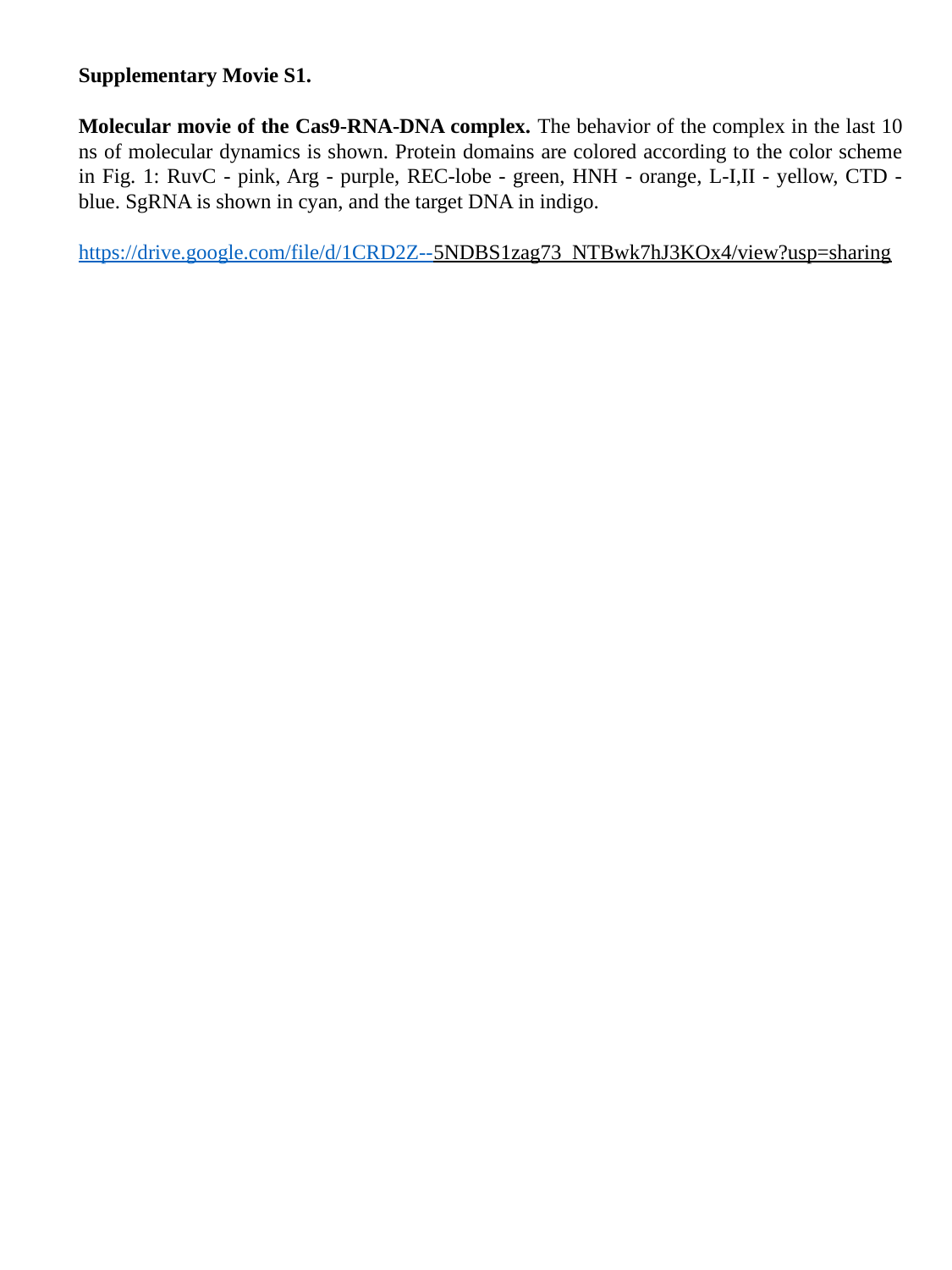

Supplementary Movie S1.
Molecular movie of the Cas9-RNA-DNA complex. The behavior of the complex in the last 10 ns of molecular dynamics is shown. Protein domains are colored according to the color scheme in Fig. 1: RuvC - pink, Arg - purple, REC-lobe - green, HNH - orange, L-I,II - yellow, CTD - blue. SgRNA is shown in cyan, and the target DNA in indigo.
https://drive.google.com/file/d/1CRD2Z--5NDBS1zag73_NTBwk7hJ3KOx4/view?usp=sharing

## Slide 3
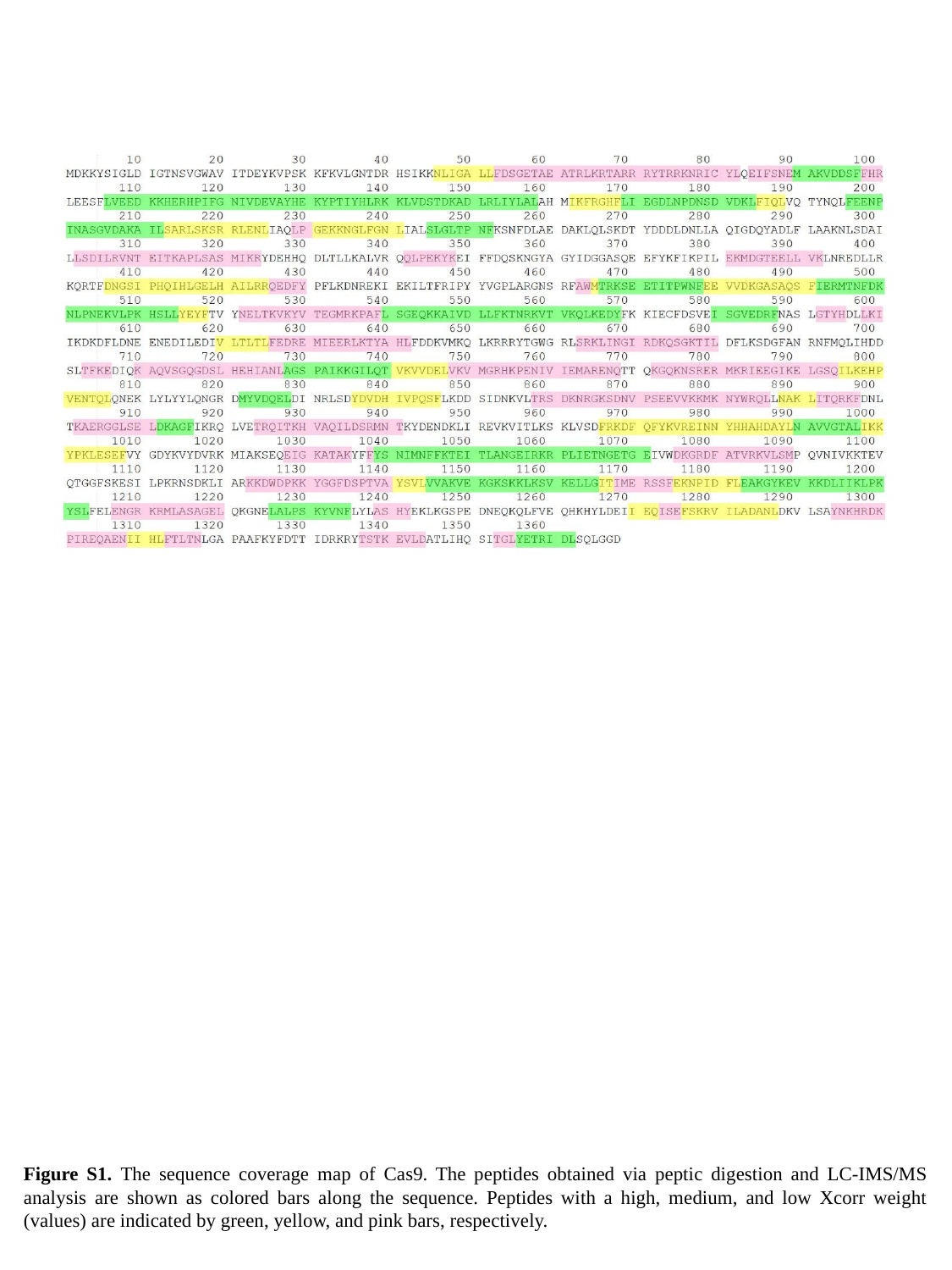

Figure S1. The sequence coverage map of Cas9. The peptides obtained via peptic digestion and LC-IMS/MS analysis are shown as colored bars along the sequence. Peptides with a high, medium, and low Xcorr weight (values) are indicated by green, yellow, and pink bars, respectively.

## Slide 4
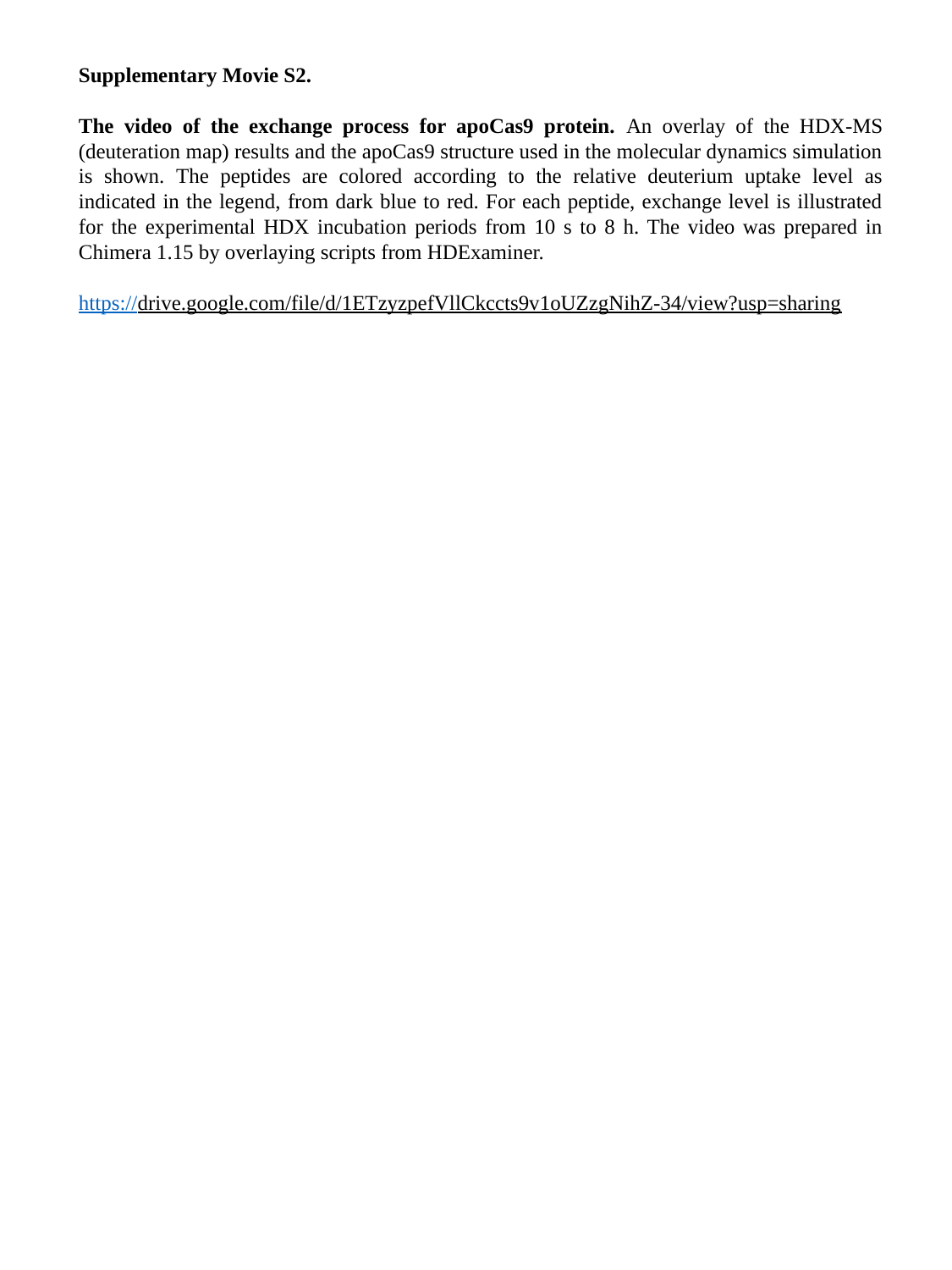

Supplementary Movie S2.
The video of the exchange process for apoCas9 protein. An overlay of the HDX-MS (deuteration map) results and the apoCas9 structure used in the molecular dynamics simulation is shown. The peptides are colored according to the relative deuterium uptake level as indicated in the legend, from dark blue to red. For each peptide, exchange level is illustrated for the experimental HDX incubation periods from 10 s to 8 h. The video was prepared in Chimera 1.15 by overlaying scripts from HDExaminer.
https://drive.google.com/file/d/1ETzyzpefVllCkccts9v1oUZzgNihZ-34/view?usp=sharing

## Slide 5
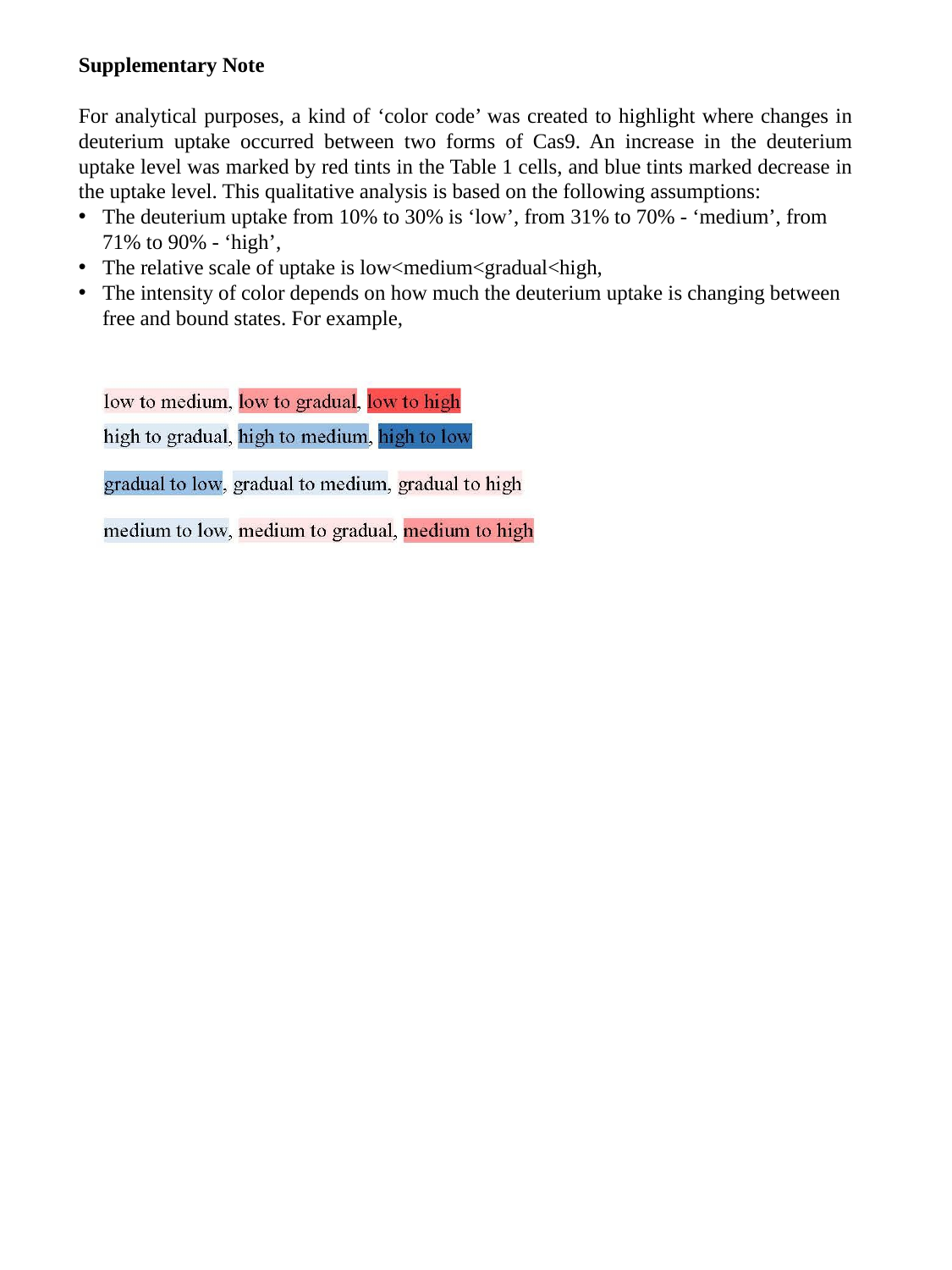

Supplementary Note
For analytical purposes, a kind of ‘color code’ was created to highlight where changes in deuterium uptake occurred between two forms of Cas9. An increase in the deuterium uptake level was marked by red tints in the Table 1 cells, and blue tints marked decrease in the uptake level. This qualitative analysis is based on the following assumptions:
The deuterium uptake from 10% to 30% is ‘low’, from 31% to 70% - ‘medium’, from 71% to 90% - ‘high’,
The relative scale of uptake is low<medium<gradual<high,
The intensity of color depends on how much the deuterium uptake is changing between free and bound states. For example,

## Slide 6
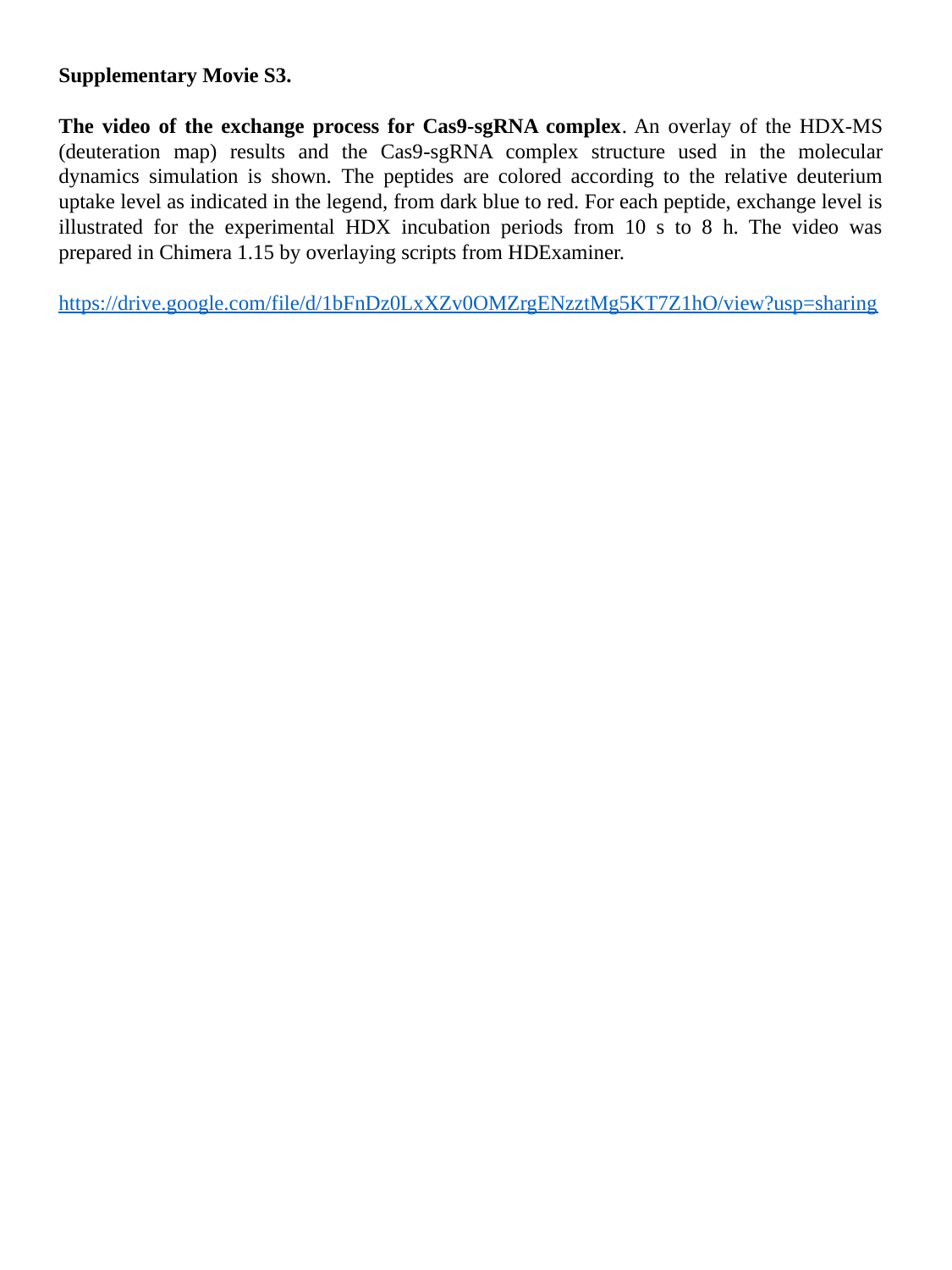

Supplementary Movie S3.
The video of the exchange process for Cas9-sgRNA complex. An overlay of the HDX-MS (deuteration map) results and the Cas9-sgRNA complex structure used in the molecular dynamics simulation is shown. The peptides are colored according to the relative deuterium uptake level as indicated in the legend, from dark blue to red. For each peptide, exchange level is illustrated for the experimental HDX incubation periods from 10 s to 8 h. The video was prepared in Chimera 1.15 by overlaying scripts from HDExaminer.
https://drive.google.com/file/d/1bFnDz0LxXZv0OMZrgENzztMg5KT7Z1hO/view?usp=sharing

## Slide 7
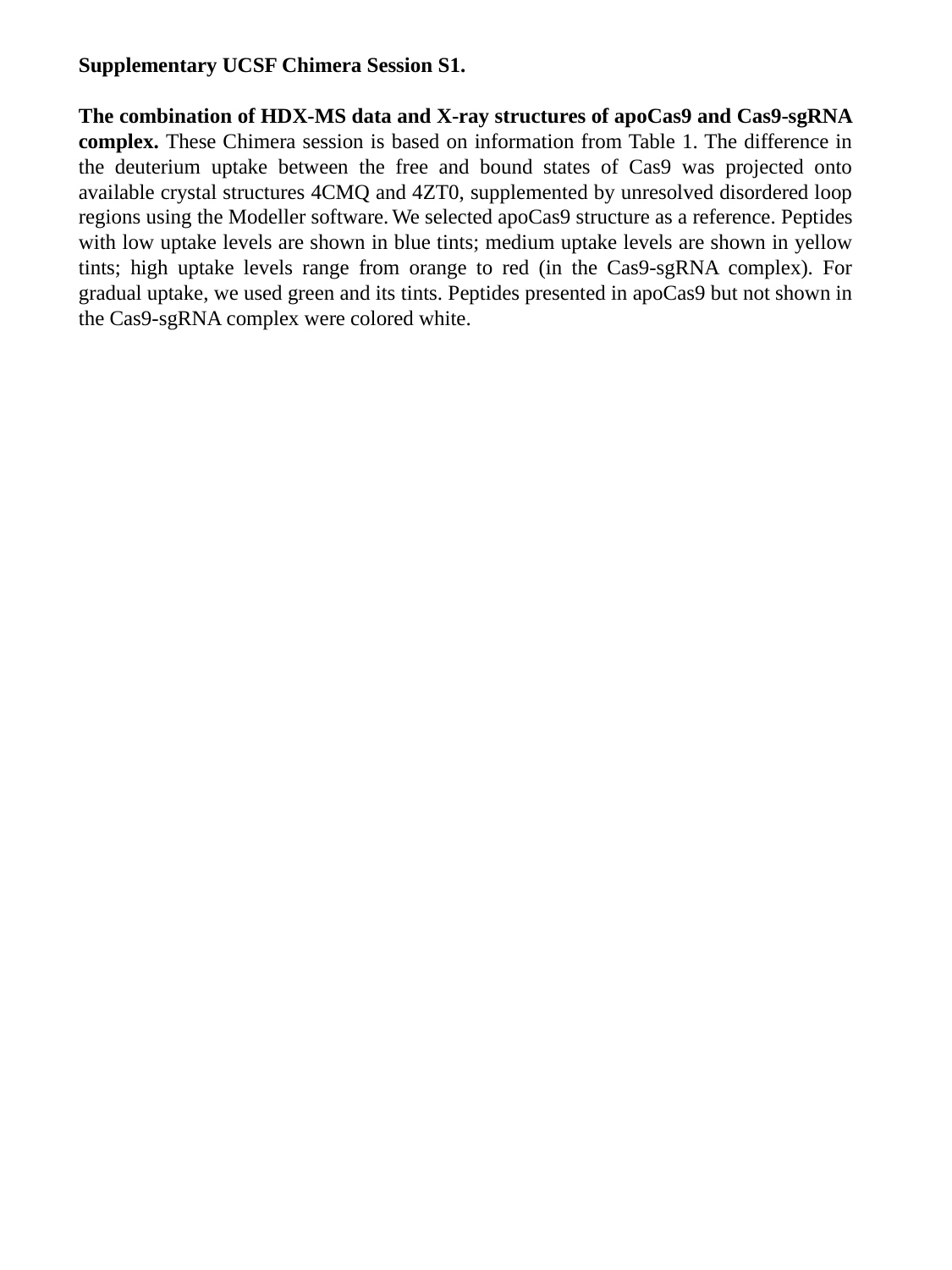

Supplementary UCSF Chimera Session S1.
The combination of HDX-MS data and X-ray structures of apoCas9 and Cas9-sgRNA complex. These Chimera session is based on information from Table 1. The difference in the deuterium uptake between the free and bound states of Cas9 was projected onto available crystal structures 4CMQ and 4ZT0, supplemented by unresolved disordered loop regions using the Modeller software. We selected apoCas9 structure as a reference. Peptides with low uptake levels are shown in blue tints; medium uptake levels are shown in yellow tints; high uptake levels range from orange to red (in the Cas9-sgRNA complex). For gradual uptake, we used green and its tints. Peptides presented in apoCas9 but not shown in the Cas9-sgRNA complex were colored white.

## Slide 8
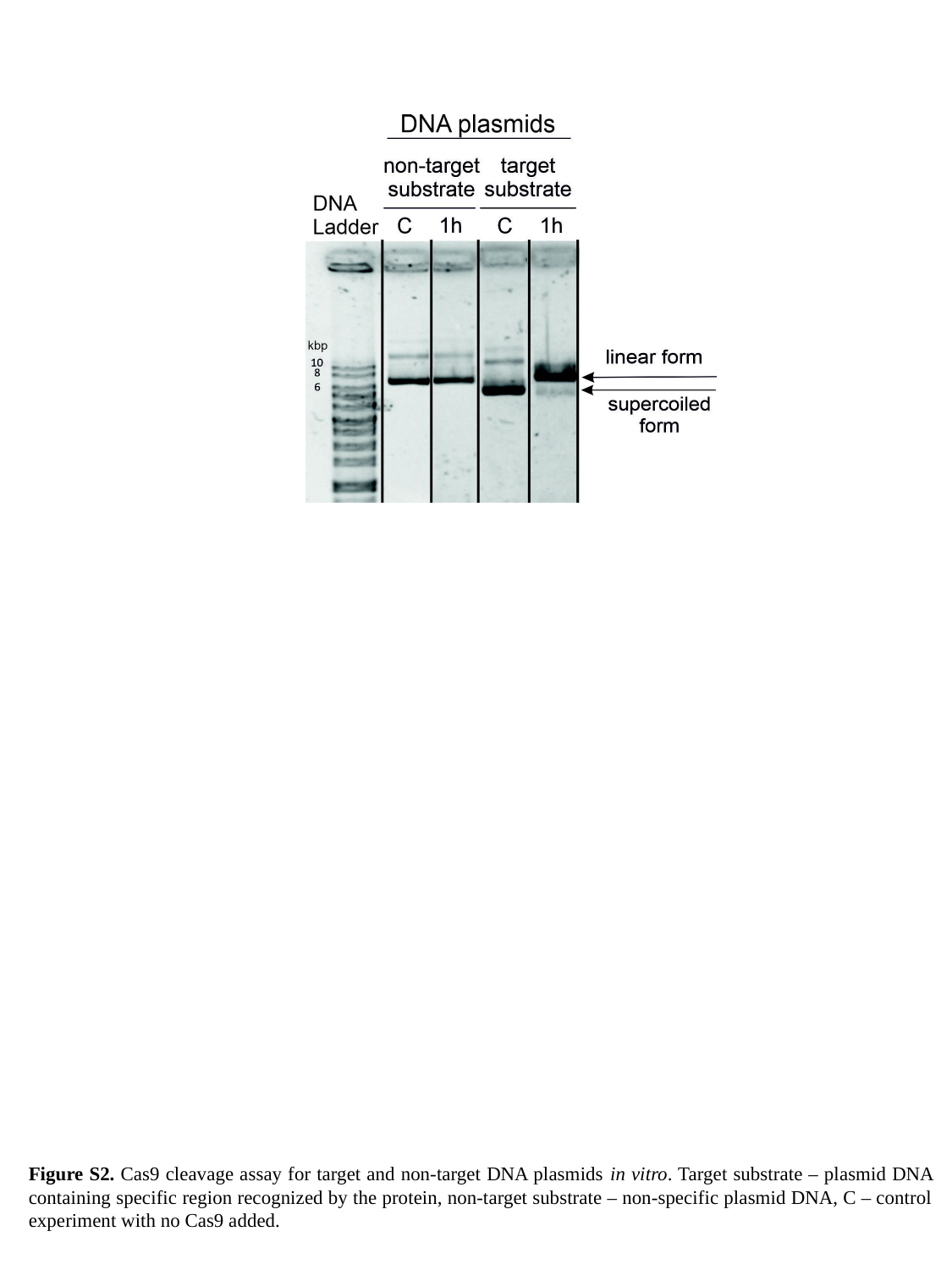

Figure S2. Cas9 cleavage assay for target and non-target DNA plasmids in vitro. Target substrate – plasmid DNA containing specific region recognized by the protein, non-target substrate – non-specific plasmid DNA, C – control experiment with no Cas9 added.
