# Supplementary figures and images for: Probing the Dynamics of Streptococcus pyogenes Cas9 Endonuclease Bound to the sgRNA Complex Using Hydrogen-Deuterium Exchange Mass Spectrometry
Source: Int J Mol Sci. 2022 Jan 20;23(3):1129. doi: 10.3390/ijms23031129 (PMC8834707; doi:10.3390/ijms23031129)

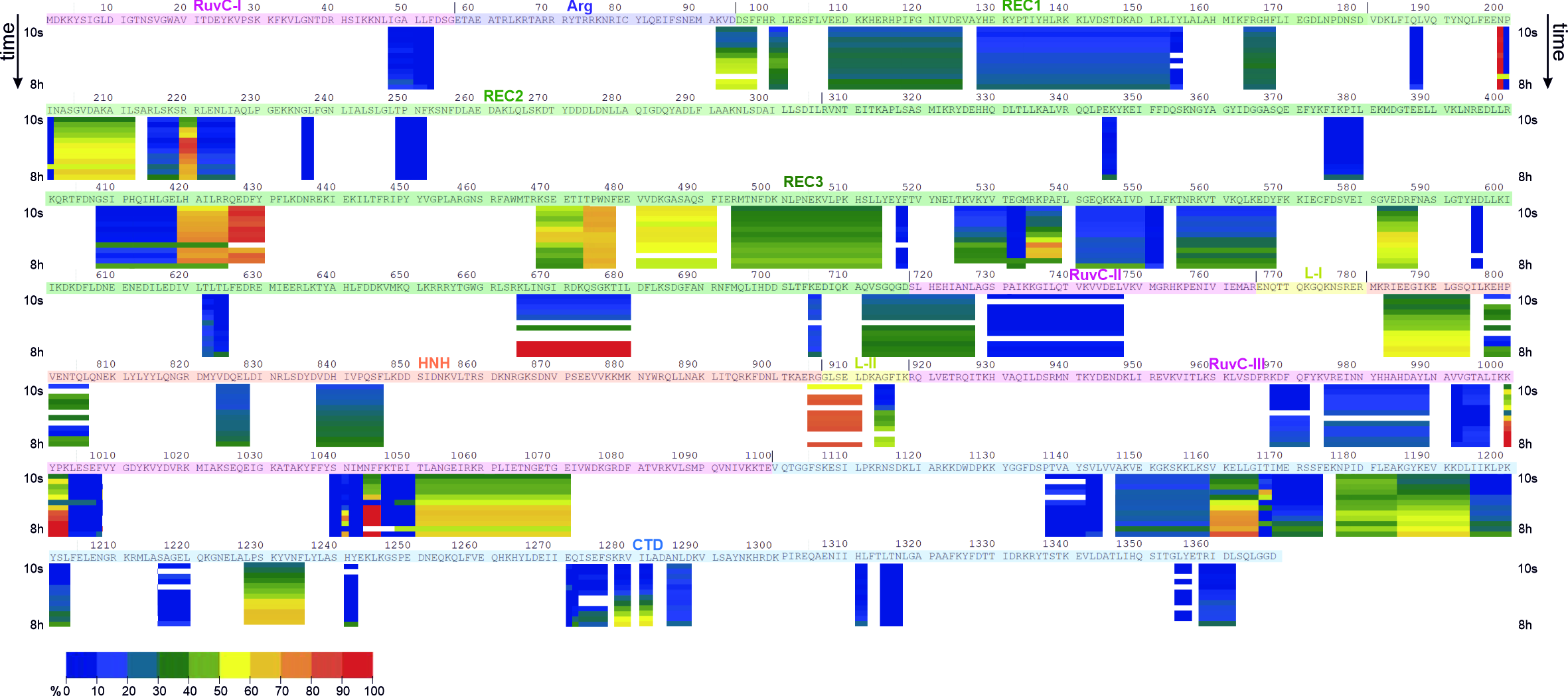

Supplement: Supplementary file 1 [file ijms-23-01129-s001.zip › Figure S4.tif]

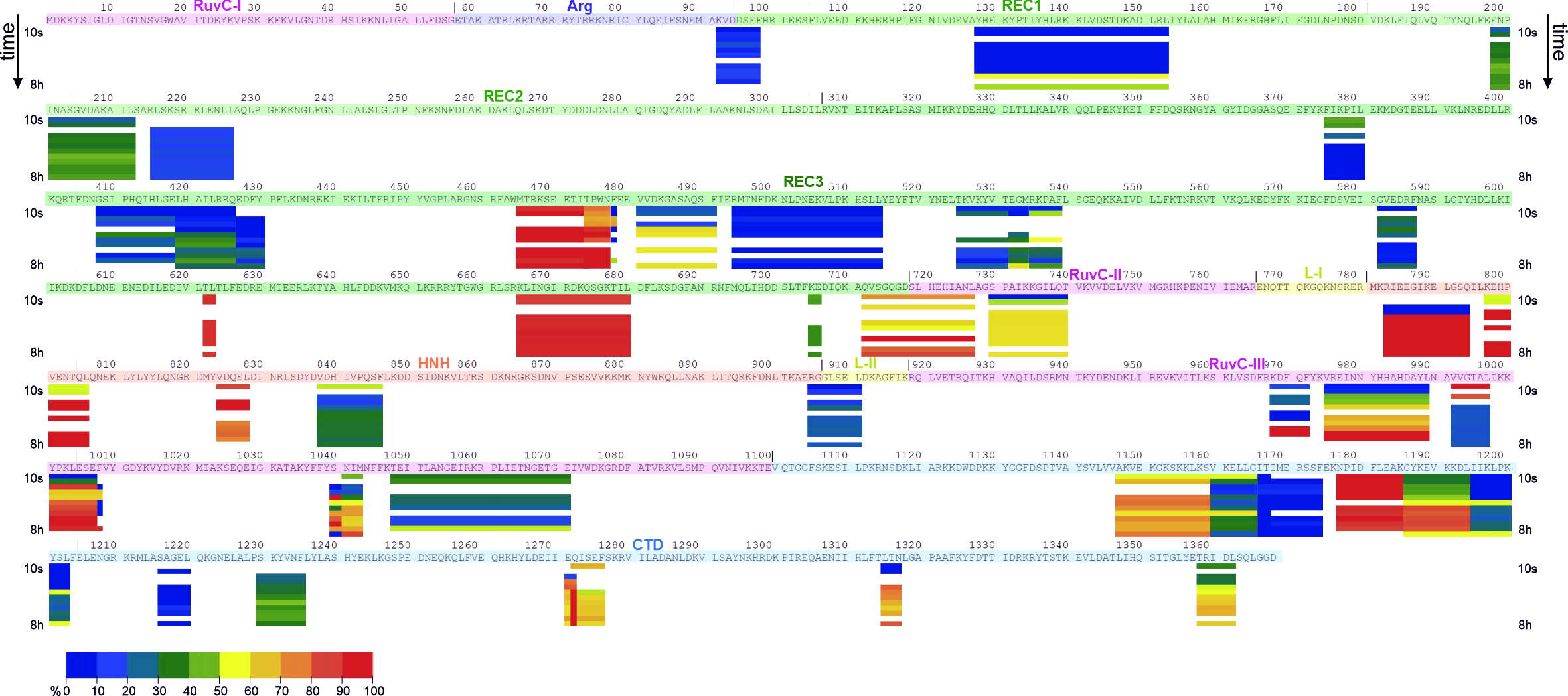

Supplement: Supplementary file 1 [file ijms-23-01129-s001.zip › Figure S5.tif]
